# 

**Published:** 1891-12

**Authors:** 


CONTENTS.

VOL. 38. NO. 12.

| | PAGE. |
|---|---|
| Special | 265 |
| The Next Volume | 265 |
| Habits of Eating | 266 |
| The Waste of the Household | 268 |
| Oatmeal as a Food | 269 |
| Overstrung Nerves | 270 |
| Food for Dyspeptics | 270 |
| Celery | 271 |
| The Medicinal Value of Onions | 272 |
| An Insect Worth Millions | 273 |
| Wedding Rings | 273 |
| The Earth's Heat | 274 |
| A Hint on Economy | 275 |
| Intense Brilliancy of Lightning | 276 |
| Bear Skin | 276 |
| Cleaning Windows and Paint | 277 |
| In Contagious Diseases | 277 |
| Care of Clothing | 278 |
| Good to Remember | 279 |
| Infant Diet | 280 |
| Miscellaneous | 281 |
| Literary | 283 |
| H——to Annie | 284 |

INDEX TO ADVERTISEMENTS OF HALF A PAGE AND OVER.

| | PAGE. |
|---|---|
| A Christmas Gift | I |
| Lydia E. Pinkham Med. Co. | II |
| Erie Medical Co. | III |
| Bovinine | IV |
| Dr. J. C. Ayer & Co | V |
| Cornish & Co | VIII |
| Hall's Journal Premiums | IX |
| Rochester Lamp Co. | X |
| Royal Baking Powder | XI |
| Herba Vitæ | XI |

